# Forensic Validity of the Third Molar Maturity Index (*I*_3M_) for Age Estimation in a Russian Population

**DOI:** 10.1155/2020/6670590

**Published:** 2020-12-11

**Authors:** Roberto Scendoni, Galina V. Zolotenkova, Stefano Vanin, Yuri I. Pigolkin, Roberto Cameriere

**Affiliations:** ^1^Department of Law, Institute of Legal Medicine (AgEstimation Project), University of Macerata, Macerata, Italy; ^2^Department of Forensic Medicine, Federal State Autonomous Educational Institution of Higher Education I.M. Sechenov First Moscow State Medical University of the Ministry of Health of the Russian Federation (Sechenov University), 119991 Moscow, Russia; ^3^Federal State Budgetary Scientific Institution, Developmental Information Technologies Center, The Russian Academy of Sciences (DITS), Odintsovo, 143003 Moscow Region, Russia; ^4^Department for the Earth, Environment and Life Sciences, University of Genova, Genova, Italy

## Abstract

The aim of this cross-sectional study was to test the accuracy of the third molar maturity index (*I*_3M_) cut-off value (0.08) to distinguish between individuals above and below the adult age of legal responsibility (18 years) in a Russian population. A sample of 571 digital panoramic radiographs of healthy Russian minors and young adults (363 females and 208 males), aged between 14 and 24 years, was evaluated. The lower left third molars were analyzed by applying the cut-off value of 0.08 determined by Cameriere et al. (2008). Lin's concordance correlation coefficient (*ρ*_c_) and Cohen's kappa coefficient (*κ*) showed that repeatability and reproducibility are high for both intra- and interobserver errors. The *I*_3M_ value decreased with age in both sexes. Age distribution gradually decreases as *I*_3M_ increases in both girls and boys. In the male group, molar maturity stages 0-0.04, 0.04-0.08, 0.08-0.3, 0.3-0.5, and 0.9-1.4 were reached slightly earlier than in the female group. The results demonstrated that sensitivity is 0.96 in boys and 0.93 in girls; associated specificity values were both 0.98. The cut-off value of *I*_3M_ is statistically robust and thus valid for forensic application in a Russian population to determine whether or not a subject has reached 18 years of age. Finally, we compared our results with those of other studies in which the same *I*_3M_ cut-off value was tested on different populations. The method is novel as it is reliable and easily reproducible, thus ensuring a universal way of comparing the results obtained (based on a cut-off value) among many populations, in order to develop an ever-increasing database.

## 1. Introduction

Today, more than ever, it is of vital importance to be able to establish a person's biological age in a number of countries. One of the main reasons for this is related to migration issues and the identification of refugees. From January to April 2020, more than 3.4 million migrants were officially registered in the Russian Federation [1]. However, a considerable number of migrants avoid registration for various reasons, including criminal motives. The fact that migrants often lack documents complicates the work of the deportation service, and the problem is compounded when it comes to borderline situations where it is unknown whether or not an individual has reached the age of majority. Foreign citizens and stateless persons often reach the territory of the Russian Federation accompanied by minors, but many young migrants between the ages of 14 and 18 arrive in Russia illegally without adults. This leads to negative consequences: they can become involved in criminal activities, illegally engaged in work, and subjected to sexual violence. There is currently no system for registering underage migrants in the Russian Federation: information about these minors is not integrated into the state migration information system. This leads to the deliberate concealment of personal information, especially age data, in order to avoid responsibility for criminal acts. Russian minors who commit criminal offenses also hide their age, for the same purpose—evasion of responsibility. Establishing the age of majority is important in both cases; it is also crucial in dealing with cases of sexual violence, since, according to the criminal code of the Russian Federation, violence against a minor increases responsibility [2].

Appropriate recommendations have been developed to determine the age of children [3, 4]. Certain methods have their own indications and limitations. The standard methodology includes an anthropometric examination, an assessment of puberty, X-ray examination of the left hand (in later cases, the clavicle), and an assessment of dental status. The most frequently performed technique is radiography of the left wrist using the Greulich and Pyle (GP) and Tanner and Whitehouse (TW) methods [5, 6]. To date, the validity of using these methods in different populations is questionable [7]. One of the significant disadvantages of hand-wrist radiograph analysis is the standard error of 2-3 years. For legal practice, accurate age determination is fundamentally important, since a number of courts do not accept age intervals. In such circumstances, the approach to determining the biological age of a person can be supplemented by methods of forensic dentistry (forensic odontology).

Dental status is used worldwide as a predictor for estimating the age of the living and the dead [8–11]. Cameriere et al. (2008) [12] proposed a method for the discrimination between adults or minors, based on the correlation between the age and the normalized measures of the open apices and height of the third molar or the third molar maturity index (*I*_3M_); the method is based on the analysis of the development of the third molar at one side of the mandible.

The progressive closure of the apices is based on prerequisites linked to dental anatomy and development. In immature teeth, root formation is incomplete; the pulp therefore communicates with the surrounding periapical tissues through a large opening; slowly, with the lengthening of the root and the deposition of dentin and cement, the apical opening is progressively reduced, and the vessels entering and exiting decrease in number. Measurements that are performed on X-ray images can significantly reduce operator-dependent error, thanks to the selection of specific drafting software. Thus, the principal advantage of this new approach is the absence of subjective assessment of the stage and degree of development of a morphological trait. With the *I*_3M_, the chronological age of an adolescent or young adult can be accurately estimated, and the *I*_3M_ threshold value < 0.08 classifies (differentiates) individuals over 18 years of age.

Going forward, the intention is to evaluate the applicability of this method in different populations. Sufficient data on the morphological parameters of the third molar development in populations around the world would increase the accuracy and reproducibility of this method. In addition, such a database would also allow the method to be adapted for use in different countries, helping scientific experts worldwide to more effectively solve legal and forensic aspects in the practice of personal identification.

The aim of this study was to test the validity of the *I*_3M_ cut-off value of <0.08 as a reliable tool for discriminating between minors and adults (i.e., over 18 years old) in a large sample of the Russian population.

## 2. Materials and Methods

A total of 571 orthopantomograms (OPGs) performed between 2014 and 2020 were obtained from the archival materials of the Clinical Center of the Institute of Dentistry of Sechenov University (Moscow, Russia). Patients of Russian nationality of three generations were selected; they lived in their birthplace in the Central European part of Russia (citizens of Moscow, Tula, Yaroslavl, and its regions). Information about nationality was clarified by the patients themselves, their parents, and municipal institutions. According to ethical and deontological principles, OPGs were collected with their consent. The study used depersonalized data, thereby eliminating the possibility of identifying a single patient.

The sex and true age of all subjects were known at the time of the study, and the OPG images showed clearly distinguishable and measurable third molars. Exclusion criteria were malformations of the teeth, hypodontia, and the presence of systemic diseases and orthodontic treatment that could affect the degree of development of the dental system, including third molars. The patient's identification number, sex, date of birth, and date of X-ray were recorded. The chronological age of the patients was recorded in decimal years. The distribution by age and sex is described in detail in Table 1.

OPGs and related measurements were performed by a forensic medical examiner and an odontologist, both specialists in radiology with at least fourteen-year experience in evaluating X-ray images for both clinical and forensic purposes.

**Figure 1:**
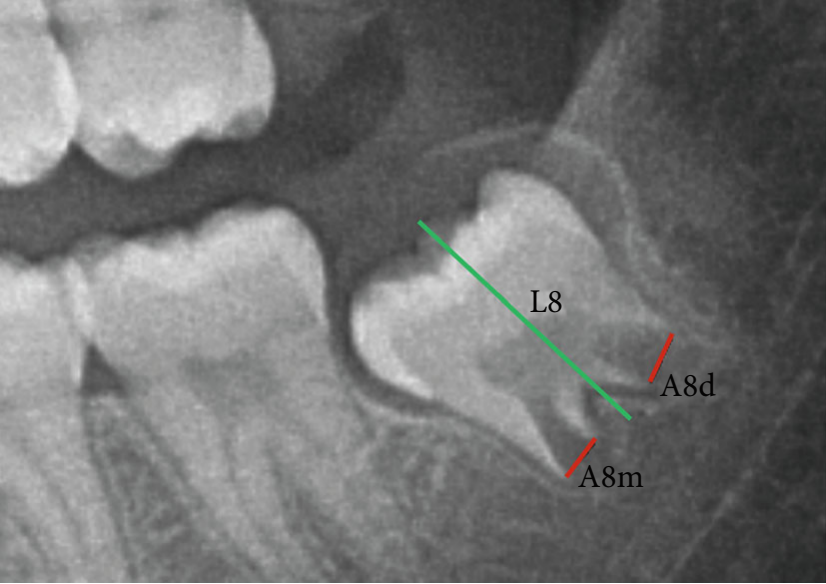
Measurements of tooth length (*L*_8_) (green line) and widths of open root apices (*A*_8m_, *A*_8d_) (red lines).

The study was based on the analysis of the lower left third molar using Cameriere et al.'s method [12]. The OPGs were exported to JPEG images, and the measurements were performed using the computer program ImageJ (Graphics Suite, Ottawa, Canada). Apices and tooth length of the lower left third molar were measured (Figure 1). The apical ends of the roots (mesial and distal) of the lower third molar were analyzed, i.e., the sum of the widths of open apices (*A*_8_), mesial (*A*_8m_), and distal (*A*_8d_); *A*_8_ measurements were normalized by dividing by tooth length (*L*_8_). So, *I*_3M_ was calculated according to the following formula:

(1) $$\begin{array}{c} I_{3\text{M}}=\frac{A_{8\left(\text{m}\right)}+A_{8\left(\text{d}\right)}}{L_{8}}. \end{array}$$

In cases where the development of the third molar was complete, the *I*_3M_ value was written as “0.”

### 2.1. Statistical Analysis

Intra- and interrater agreement was evaluated by Lin's concordance correlation coefficient (*ρ*_c_) and Cohen's kappa coefficient (*κ*). In particular, 100 panoramic radiographs were randomly selected six weeks after the initial scoring process. Tables were used to show the relationships between chronological age and *I*_3M_ values for both sexes. A two-way contingency table was created in order to assess the performance of the specific cut-off value of *I*_3M_, and the 95% confidence interval was also applied to reveal the uncertainty associated with the estimates. The accuracy of the test was assessed by its sensitivity (i.e., the proportion of subjects older than or equal to 18 years of age with *I*_3M_ < 0.08) and its specificity (i.e., the proportion of individuals younger than 18 with *I*_3M_ ≥ 0.08). Positive predictive values (PPV), negative predictive values (NPV), and positive and negative likelihood ratios (LR+ and LR−) were also calculated to determine the probability that a positive screening result (*I*_3M_ < 0.08) truly reflects the correct age of the individual (subjects older than or equal to 18 years of age). The greater the value of the likelihood ratio for a positive test result, the more likely a positive screening is a true positive (i.e., the subject's biological age is 18 years or older). According to Bayes' theorem, posttest probability was written as

(2) $$\begin{array}{c} \text{PPV}=p_{0}\times \frac{\text{sensitivity}}{\left(\;p_{0}\times \text{sensitivity}\right)\;}+\left(1-p_{0}\right)\times \left(1-\text{specificity}\right), \end{array}$$

where *p*_0_ is the pretest probability of being 18 years or older.

## 3. Results

Average values of the true and calculated ages for males and females are shown in Table 2.

The *I*_3M_ value decreased with age in both sexes. In the male group, molar maturity stages 0-0.04, 0.04-0.08, 0.08-0.3, 0.3-0.5, and 0.9-1.4 were reached slightly earlier than in the female group.

The *I*_3M_ value was calculated separately for males and females. Clear sex differences were revealed by a comparison of the data obtained, and the test results for both sex groups are shown in Tables 3(a) and 3(b).

In the analysis of the results, *I*_3M_ value > 0.08 was mostly observed in males under 18 years (80 cases) compared to the over 18 age group (5 cases) (PPV 98.4%, NPV 94.1%). Thus, the sensitivity of the test in the male group was 0.96, and the specificity of the test was 0.98 (Table 4).

In the female group, the *I*_3M_ value > 0.08 was noted in 139 cases in persons under 18 years and in 15 cases in persons over 18 years. At *I*_3M_ < 0.08, the age was more than 18 years in 206 cases and in 3 cases, the age was less than 18 years (PPV 98.6%, NPV 90.2%). The specificity of the test was 0.98, and the sensitivity of the test was 0.93 (Table 4).

## 4. Discussion

Forensic medical experts are involved whenever it is necessary, from a legal perspective, to determine whether an individual is a minor or an adult (18 years or above). To assess the age of children and adolescents, X-ray studies are used in accordance with the recommendations of AGFAD [13, 14]. Orthopantomograms are a crucial element in determining whether a person is under 18 years of age [13]. Panoramic radiography in forensic dentistry makes it possible to measure the third molar and estimate age using the method proposed by Cameriere et al. [12]. This tooth type is chosen because in the adolescent period, most teeth have already formed, and only the third molar requires a few more years of root system development, which makes it useful as an indicator (delimiter) for assessing 18 years of age (older or younger) [15–22].

The trend of modern research is to create formulas and methods for age estimation that generate the most accurate results and then to adapt them to individual populations. This reduces the likelihood of errors in the forensic examination.

The *I*_3M_ value (cut − off = 0.08) was recently tested in many populations around the world. The results obtained demonstrated the high sensitivity and specificity of Cameriere et al.'s method, while the percentages were different [12, 16–22]. Based on a systematic review and meta-analysis, Haglund and Mörnstad (2019) concluded that the analysis of third molar development using dental X-rays is one of the most commonly used methods of forensic age assessment. All studies (82) showed a low level of false-positive results when determining ≥18 years of age using the maturity stages of third molars [23]. However, these studies did not include the Russian Federation. The purpose of the current study was therefore to evaluate the effectiveness of using a *I*_3M_ cut-off value of 0.08 for this particular population.

In fact, the Russian Federation consists of 190 nationalities. According to the all-Russian population census (2010) (a dataset provided by Rosstat), Russians make up almost 77% of the total. At the same time, the population of North Caucasians has increased from 1 to 5% over the past 10 years, according to the available population census data from 2002 to 2010 [24]. This confirms the need for further interethnic research on the territory of the Russian Federation.

The question of interpopulation differences (and the reasons why they occur) remains debatable. Over the past few decades, extensive research has been conducted on variations in the anatomy of human teeth, in an attempt to establish relationships to sex, ethnicity, and race. Research spanning the last century has demonstrated that there are certain trends in the distribution of absolute sizes of teeth by racial groups [25–28]. Differences have been found between racial groups with regard to molar crown indices, specifically the ratio between the buccolingual and mesial-distal diameters. This index, for example, in Caucasians, is usually very high for the second and third upper molars. In the early 1970s, an “odontographic map of the world” was suggested [29].

More recently, Olze et al. (2007) conducted a comparative study and found differences in the teething process between three ethnic groups. The authors concluded that population specificity must be taken into account when assessing age and that there is a need to expand the reference data for this purpose in forensic medical examination [30]. However, Thevissen et al. (2010) did not find statistically significant differences in the dynamics of the third molar development between populations of different countries [15]. Some modern researchers believe that the growth and development of teeth, including the third molar, is genetically determined. Others point to the relationship between dental underdevelopment and low socioeconomic status, due to lack of nutrition [19].

As in previous studies conducted in Albania [16], Australia [17], Italy [12, 18], Colombia [19], Egypt [20], and other countries, measurements of the third molar were performed during this research project using a single scheme: the *I*_3M_ was determined and the accuracy (sensitivity and specificity) of the test was verified. Statistical analysis of the data obtained from the Russian population found that the results are generally similar to previous studies, but with some noticeable differences.

In a study by Cameriere et al. [16] on an Albanian population, the sensitivity of the *I*_3M_ test was 94.1% (0.94) in men and 75.4% (0.75) in women, and the specificity was 90.9% (0.90) in men and 96.6% (0.96) in women. In Australia, the results demonstrated a sensitivity of 90% in both sexes and specificity was 0.85 in men and 0.87 in women [17].

As regards the population of Sardinia (Italy), accuracy in the male group was 0.87 and sensitivity 0.85, and in the female group, accuracy was 0.84 and sensitivity 0.79 [18]. In research on a Columbian sample, the test results for the male group showed a sensitivity of 91.7% (0.91), with a specificity of 90.6% (0.90); for the females, these values were 95.1% (0.95) and 93.8% (0.93), respectively [19]. The percentage of correctly classified ages was 89.7%, which is comparable to similar studies.

In the Egyptian population, the sensitivity of the test for men was 0.95, with a specificity of 100%; for women, 73% (0.73) sensitivity and 97% (0.97) specificity were demonstrated. In this sample, the percentage of correctly classified individuals was 97% for men (higher than all previous studies) and 82% for women [20]. In the Turkish population, for the male group, the sensitivity was 94.6% (0.94) with a specificity of 100%; in females the sensitivity was 85.9% (0.85) and the specificity was 100%, while the percentages of correct answers were 92.7% and 97.6%, respectively [21]. In the French population, sensitivity and specificity were 0.813 and 0.962 for women and 0.871 and 0.953 for men, respectively. The classification accuracy was 0.897 for women and 0.916 for men [22]. Sensitivity and specificity in most studies were higher in the male sample.

Our study showed that in the Russian population, the method demonstrated a greater sensitivity and specificity when compared with the abovementioned studies, providing a more accurate forecast. By comparing the available data with previous results, we can conclude that for each population, the age range is distinguished according to the most noticeable changes in the degree of mineralization and maturity of teeth. However, in practice, this is expressed in a single pattern: with increasing age, the *I*_3M_ decreases in all groups. For each group, a threshold value is defined, which is crucial in determining whether a person is under or over 18 (Table 5).

In our study, the age at *I*_3M_ = 0.08 was close to the values of the French population in both the male and female groups. Time-lapse analysis of dental maturity in modern population samples showed clear differences in the onset of maturity (mineralization) of the third molar. This confirms the need to develop ethnically based databases for age assessment.

When comparing sex differences, the female groups representative of Australia, Italy, France, and Russia showed earlier tooth maturation. As in previous studies, we found that the test classifies men more accurately (the percentage of correct results is higher) than women.

## 5. Conclusions

The *I*_3M_ is an X-ray anatomical index that provides valuable information pertaining to different ethnic groups. The analytical method used has a significant correlation coefficient compared to other less common dental techniques and is optimal and accurate for age identification, making it a useful tool for discriminating between people over and under 18 years of age. Thus, the results of previous studies and the results of this work confirm the high specificity and sensitivity of the *I*_3M_ for determining the age of 18. This proves that it can be successfully applied to determine the age in many forensic contexts, such as personal identification in the aftermath of mass disasters or air crashes, or for age estimation in the interests of imputability, adoption procedures, protection of unaccompanied minors, etc. Furthermore, it is of paramount importance to apply this method to large samples of other populations in order to improve its accuracy and contribute towards solving the puzzle of “ethnic identity,” given that populations belonging to different ethnic groups may show more similar anthropometric indices and dental development than subpopulations of the same ethnic group.

## Figures and Tables

**Table 1:** Sample of panoramic radiographs obtained from a Russian population according to sex and age categories.

Age (years)	Females	Males	Total
14	41	24	65
15	35	21	56
16	36	21	57
17	29	16	45
18	26	9	35
19	32	13	45
20	40	26	66
21	31	19	50
22	48	19	67
23	38	26	64
24	7	14	21
Total	363	208	571

**Table 2:** Summary statistics of chronological age according to third molar maturity index (*I*_3M_): number of individuals (*N*), average (AVG), mean standard deviation (SD), minimum value (MIN), median (MED), and maximum value (MAX).

	*N*	AVG	SD	MIN	MED	MAX
Females						
0.0-0.04	130	21.4	1.5	19	21.8	24.9
0.04-0.08	79	20.5	2.0	17	16	24.4
0.08-0.3	56	17.0	1.8	14	16	23.0
0.3-0.5	29	15.5	1.0	14	15	17.1
0.5-0.7	20	15.0	1.1	14	15	17.0
0.7-0.9	25	15.0	1.2	14	15	17.4
0.9-1.4	24	14.9	0.8	14	15	16.4
Males						
0.0-0.04	84	22.1	1.7	18.8	22.1	24.8
0.04-0.08	39	20.3	1.7	16.0	20.1	24.8
0.08-0.3	40	16.5	1.1	14.0	16.5	18.7
0.3-0.5	14	14.7	0.9	14.0	14.0	16.7
0.5-0.7	9	15.2	0.7	14.3	15.1	17.0
0.7-0.9	12	15.0	1.0	14.0	14.8	16.1
0.9-1.4	10	14.6	0.7	14.0	14.5	16.0

**Table:** (a) Contingency table describing discrimination performance of the test for males (cut-off 0.08)

Age			
Test	>18	<18	Total
<0.08	121	2	123
>0.08	5	80	85
Total	126	82	208

**Table:** (b) Contingency table describing discrimination performance of the test for females (cut-off 0.08)

Age			
Test	>18	<18	Total
<0.08	206	3	209
>0.08	15	139	154
Total	221	142	363

**Table 4:** Summary of the characteristics of the test for males and females when the cut-off 0.08 is applied.

Parameter	Males	Females
Sensitivity	0.96	0.93
Specificity	0.98	0.98
LR	48.0	46.5
PPV	98.4%	98.6%
NPV	94.1%	90.2%

**Table 5:** Summary mean values of chronological age in men and women of different populations according to third molar maturity index cut-off *I*_3M_ = 0.08.

Population	Average age of *I*_3M_ = 0.08 in males (years)	Average age of *I*_3M_ = 0.08 in females (years)
Colombia [19]	14.50 ± 0.6	14.52 ± 0.43
France [22]	17.04 ± 1.34	16.81 ± 1.42
Albania [16]	17.1 ± 1.2	17.6 ± 1.2
Italy [12]	17.23 ± 2.30	17.14 ± 2.15
Turkey [21]	17.32 ± 1.59	16.54 ± 1.4
Australia [17]	17.56 ± 2.06	17.23 ± 2.27
Sardinia [18]	18.29 ± 1.69	17.48 ± 0.97
Russia (current research)	16.5 ± 1.1	17.0 ± 1.8

## Data Availability

Data were obtained and are available from the archive material of the Clinical Center of the Institute of Dentistry of Sechenov University.
